# Interactions of Human Dermal Dendritic Cells and Langerhans Cells Treated with *Hyalomma* Tick Saliva with Crimean-Congo Hemorrhagic Fever Virus

**DOI:** 10.3390/v10070381

**Published:** 2018-07-20

**Authors:** Sergio E. Rodriguez, Alexander J. McAuley, Aysen Gargili, Dennis A. Bente

**Affiliations:** 1 Department of Microbiology & Immunology, Galveston National Laboratory, University of Texas Medical Branch, Galveston, TX 77555-0610, USA; seerodri@UTMB.EDU (S.E.R.); Alex.Mcauley@csiro.au (A.J.M.); agargili@yahoo.com (A.G.); 2 Department of Basic Medical Sciences, Marmara University, 34722 Kadiköy/Istanbul, Turkey

**Keywords:** Crimean-Congo hemorrhagic fever virus, Crimean-Congo hemorrhagic fever, *Hyalomma marginatum*, human cutaneous immune response, Langerhans cells, dermal dendritic cells, tick-borne virus, tick-virus-host interface

## Abstract

Crimean-Congo hemorrhagic fever virus is one the most important and wide spread tick-borne viruses. Very little is known about the transmission from the tick and the early aspects of pathogenesis. Here, we generate human cutaneous antigen presenting cells—dermal dendritic cells and Langerhans cells—from umbilical cord progenitor cells. In order to mimic the environment created during tick feeding, tick salivary gland extract was generated from semi-engorged *Hyalomma marginatum* ticks. Our findings indicate that human dermal dendritic cells and Langerhans cells are susceptible and permissive to Crimean-Congo hemorrhagic fever virus infection, however, to different degrees. Infection leads to cell activation and cytokine/chemokine secretion, although these responses vary between the different cell types. *Hyalomma marginatum* salivary gland extract had minimal effect on cell responses, with some synergy with viral infection with respect to cytokine secretion. However, salivary gland extract appeared to inhibit antigen presenting cells (APCs) migration. Based on the findings here we hypothesize that human dermal dendritic cells and Langerhans cells serve as early target cells. Rather affecting Crimean-Congo hemorrhagic fever virus replication, tick saliva likely immunomodulates and inhibits migration of these APCs from the feeding site.

## 1. Introduction

Crimean-Congo hemorrhagic fever (CCHF) is a viral, tick-borne zoonosis and is one of the high-priority pathogens identified in the World Health Organization as well as United States National Institutes of Health (NIH)/National Institute of Allergy and Infectious Diseases (NIAID) priority A list because of its high case fatality rate, its public health impact and the difficulties in treatment and prevention [1]. CCHF is widespread, now found in Europe, Asia, Africa, the Middle East, and the Indian subcontinent. The CCHF virus (CCHFV) circulates silently in its tick reservoir, ticks of the genus *Hyalomma*, and animal hosts in which it causes no overt disease [2]. Recent studies have indicated that subclinical infections in areas with high endemicity are greater than previously expected which could be attributed to strain differences [3,4,5]. Since most of the infections besides nosocomial transmission are acquired through tick bite, we hypothesize that in subclinical cases the virus is cleared early after transmission from the tick. Unfortunately, very little is known about the tick–virus–host interface and the early aspects of pathogenesis [2,6]. To elucidate this mechanism, we must identify the initial target cells. Previous studies have shown that dendritic cells (DC) and monocytes are the main cells in circulation susceptible to CCHFV infection [7,8] and can serve as target cells for other hemorrhagic fever viruses [9,10]. The infection of DCs has a dual effect: virus replication, and modulation of cellular immune functions. Two tissue-resident immune cell populations principally modulate immune activity within the skin; Langerhans cells (LC) and dermal dendritic cells (dDC) [11]. LC reside within, and sample antigens from, the epidermis, regulating tissue immune activation, specifically tolerance, and local T-cell effector functions. They also participate as antigen-presenting cells (APCs) in local draining lymph nodes [12]. dDCs, residing within the dermis, engage in migratory immune surveillance, draining lymph node antigen cross-presentation, and local T-cell subtype polarization [13,14]. Together, these cells oversee the immune status and cellular response across the entirety of the skin. Recent studies have demonstrated that viruses employ different ways to target and immunomodulate this subset of dermal APCs [15,16,17].

Tick saliva is a complex mixture serving a variety of functions including cytolytic, vasodilator, anticoagulant, anti-inflammatory, and immunosuppressive activity [18]. Co-evolution of ticks, vertebrate hosts and tick-borne pathogens has led to a phenomenon called saliva-assisted transmission (SAT) in which enhancement of transmission of tick-borne pathogens occurs by tick saliva, with the effect documented for several tick-borne pathogens [19]. However, few studies have looked functions of saliva from the genus *Hyalomma* [20,21,22] and there is only limited information that suggest SAT occurs during CCHFV transmission [23,24]. Here, we generate human dermal antigen cells (dDCs and LCs) from umbilical cord CD34+ progenitor cells. The APCs are susceptible and permissive to CCHFV infection. Surprisingly, tick salivary gland extract did not appear to enhance CCHFV infection in APCs, although it had a significant influence on the immune response of these cells.

## 2. Materials and Methods

### 2.1. Viruses and Stock Generation

CCHFV strain IbAr 10200 and AP92/P7 were kindly provided by Thomas Ksiazek (World Reference Collection for Emerging Viruses and Arboviruses, University of Texas Medical Branch, Galveston, TX, USA). IbAr 10200 had been passaged 10 times in suckling mice, once in Vero cells and two times in SW-13 cells. AP92/P7 was passaged eight times in suckling mice, once in Vero cells and once in SW-13 cells. Neither virus was plaque purified. SW-13 cells (ATCC catalog number CCL-105) passaged up to 25 times were maintained in L-15 medium containing 10% heat-inactivated fetal bovine serum (FBS), 100 mM l-glutamine, 50 U/mL penicillin, 50 μg/mL streptomycin (all from Sigma, St. Louis, MO, USA). Virus stock and inoculates tested negative for pyrogen contamination with a Pyrogent plus test kit (Lonza, Wakersville, MD, USA). Work with infectious CCHFV was performed in a biosafety level 4 (BSL-4) facility at the Galveston National Laboratory, University of Texas Medical Branch, Galveston, TX, USA.

### 2.2. Generation of Human Dermal Dendritic Cells and Langerhans Cells

**Figure 1:**
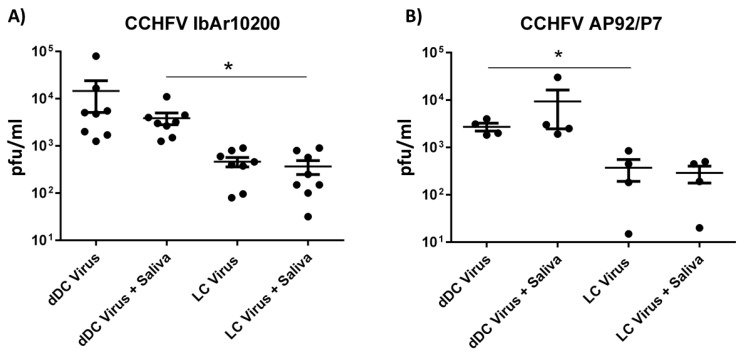
CCHFV titers in supernatant of human APCs. dDC and LC were generated from eight different donors and infected with two strains of CCHFV, IbAr10200 (**A**) and AP92/P7 (**B**), either with tick SGE (10 μg per well) or without at a MOI of 0.1. Forty-eight hours post infection supernatant was collected and virus titers determined by plaque assay. Significance between mock and indicated treatment groups at *p* < 0.05 is designated with an asterisk (*) symbol.

Langerhans and dermal dendritic cells were generated based on the protocol by Rozis et al. [25]. Briefly, umbilical cord blood samples were obtained from consented mothers in full term labor at the obstetrics and gynecology department at the University of Texas Medical Branch (UTMB) after approval was obtained from the Internal Review Board. CD34+ cells were isolated using immunomagnetic beads (STEMCELL Technologies, Vancouver, BC, Canada) and cultured in complete RPMI1640 (100 IU/mL of penicillin, 0.1 mg/mL of streptomycin, 2 mM l-glutamine; Sigma-Aldrich) with 10% fetal bovine serum (Invitrogen, Carlsbad, CA, USA) supplemented with 100 ng/mL of granulocyte-macrophage colony stimulating factor (GM-CSF), and 100 ng/mL of TNF-α (Miltenyi Biotec, Auburn, CA, USA). After 5 days, two distinct populations were present: CD14+ CD1a− and CD14−CD1a+ (Figure 1A). CD14+ cells were separated with immunomagnetic beads (STEMCELL Technologies, Vancouver, BC, Canada) and cultured separately for a further 5–7 days in GM-CSF (100 ng/mL) and IL-4 (1000 units/mL) (Miltenyi Biotec, Auburn, CA, USA). The remaining cells were cultured for the same period of time in medium supplemented with GM-CSF (100 ng/mL), TNF-α (100 ng/mL) and transforming growth factor-β (1 ng/mL) (Miltenyi Biotec, Auburn, CA, USA). Purity of generated cell populations was assessed by flow cytometry (Guava easyCyte, MerckMillpore, Burlington, MA, USA) using CD11b and CD207 (STEMCELL Technologies, Vancouver, BC, Canada) antibodies.

### 2.3. Tick Salivary Gland Extract Preparation

*Hyalomma marginatum* used in this study were collected in Yozgat state of Turkey in 2012. The strain has been maintained by standard rearing practices at the University Texas Medical Branch, Galveston, TX as previously described [26]. The ticks were tested negative for CCHFV, Tick-Borne Encephalitis Virus (TBEV), and *Rickettsia* spp. Unfed adults were placed into an ear bag on a New Zealand white rabbit (*Oryctolagus cuniculus*) and allowed to feed following protocols approved (#1209055, 31 August 2013) by the Institutional Animal Care and Use Committee of UTMB. The ticks were removed on the second day after commencement of feeding, separated by sex, surface cleaned with 70% ethanol and dissected. Salivary glands were removed and placed into sterile-filtered 0.15 M, Dulbecco’s phosphate buffered saline (Sigma, St. Louis, MO, USA), pH 7.2 held on ice. Salivary glands were sonicated at 55 kHz for 1 min on ice in a water bath. Salivary glands were pelleted by centrifugation at 10,000× *g* for 10 min and clarified salivary gland extract (SGE) was sterile filtered through a 0.22 µm Durapore-PVDF syringe filter apparatus (MerckMillipore, Burlington, MA, USA). Protein concentration was determined by Pierce BCA protein assay (Thermo, Rockford, IL, USA) and protein sizes were analyzed by BioAnalyzer (Agilent Technologies, Austin, TX, USA; see supplemental Figure S1) using an Agilent Protein 80 Kit, separated into 20 μL aliquots, subsequently frozen at −70 °C and thawed not more than twice.

### 2.4. LC and dDC Studies

For all of the experiments ca. 500,000 LCs or dDCs were used and either incubated with cell culture medium (=mock), 10 μg of *H. marginatum* SGE in cell culture medium (=SGE), CCHFV IbAr10200 at a multiplicity of infection (MOI) of 0.1 (=virus), or both 0.1 MOI CCHFV and 10 μg of *H. marginatum* SGE (=virus + SGE). For virus replication studies, cells were infected with either CCHFV strain IbAr10200 or AP92/P7 at a MOI of 0.1 and incubated for 48 h at 37 °C after which supernatant was collected. Viral titers in supernatant were measured by plaque assay as previously described [27]. For the other studies, cells were infected with CCHFV strain IbAr10200 at a MOI of 1, and incubated at 37 °C. For the gene array studies, cells were harvested 12 h post infection to determine gene expression levels. For the cytokines studies, supernatant was collected 24 h after infection.

### 2.5. Gene Array Assay

LCs and dDCs were harvested 12 h post infection and total RNA was isolated from cells by phenol/chloroform extraction based on approved BSL4 protocols. RNA quality was determined using the BioAnalyzer (Agilent Technologies, Austin, TX, USA) using an Agilent RNA 6000 Nano Kit. RNA from all samples were reverse transcribed to cDNA using the QuantiTect Rev. Transcription Kit (Qiagen, Valencia, CA, USA). The RT² Profiler™ PCR Array Human Dendritic & Antigen Presenting Cell (Qiagen, Valencia, CA, USA) was used and the samples were run on a Roche Lightcycler real-time detection system (Roche, Basel, Switzerland). RT² Profiler™ PCR Array profiles 84 related genes simultaneously and enable gene expression analysis of 170+ pathways. Data analysis was conducted using Ingenuity^®^ Pathway Analysis (Qiagen, Valencia, CA, USA).

### 2.6. Cytokine Detection

To determine secreted cytokine levels from the dDCs and LCs, supernatant was collected 24 h after infection and clarified by briefly centrifuging for 3 min at 10,000 × *g*. Twenty-microliter amounts of clarified supernatant were run in duplicates with a multiplex Milliplex MAP Human Cytokine/Chemokine kit (MerckMillpore, Burlington, MA, USA) according to the manufacturer’s instructions. The kit simultaneously quantifies granulocyte-macrophage colony-stimulating factor (GM-CSF), IFN-α, interleukin-1α (IL-1α) and -1β (IL-1β), IL-6, IL-7, IL-8, IL-10, IL-12 (p40), IL-12 (p70), IL-15, MCP-1, and tumor necrosis factor (TNF). The samples were run on a Luminex Platform (Qiagen, Valencia, CA, USA) and analyzed using Luminex 100 IS software (Luminex).

### 2.7. Migration Assay

A migration assay was performed based on the protocol by Skallova et al. [28]. Deidentified buffy coats were obtained from adult healthy donors from the UTMB Blood Bank by centrifugation enrichment with clinical approval from the UTMB Institutional Review Board. Monocytes were isolated from peripheral blood mononuclear cell (PBMC) pools using positive selection anti-CD14 monoclonal antibody coated magnetic beads according to manufacturer’s instructions (Miltenyi Biotec, Auburn, CA, USA). Monocytes were supplemented with Advanced RPMI 1640 media, 10% FBS, 2 mM l-glutamine (Invitrogen, Carlsbad, CA, USA), 1% penicillin/streptomycin (Invitrogen, Carlsbad, CA, USA), and monocytes were differentiated to monocyte derived dendritic cell (moDC) phenotype by the addition of 0.05 mM β-mercaptoethanol, 50 ng/mL granulocyte-macrophage colony-stimulating factor (GM-CSF), and 16 ng/mL interleukin-4 (IL-4) (R&D Systems, Minneapolis, MN, USA). The moDC were incubated for one week at 37 °C, 5% CO_2_, harvested with Accutase cell detachment solution (STEMCELL Technologies, Vancouver, BC, Canada), and were incubated with 1 μg/mL Lipopolysaccharide (LPS) from *Escherichia coli* O55:B5 (Sigma, St. Louis, MO, USA) was added to each well to induce moDC maturation. The moDC were pelleted by centrifugation and suspended in Roswell Park Memorial Institute (RMPI) medium with 20 ug/mL of tick SGE and placed into a polycarbonate membrane insert within a 24-well Fluorimetric QCM Cell Migration Assay (MerckMillipore, Burlington, MA, USA) migration chamber at a density of 5 × 10^5^ cell per chamber. Cells were incubated above wells containing serum-free RMPI with or without 1 μg/mL of the chemoattractant, recombinant human CCL-19 (MIP-3β) (BioLegend, San Diego, CA, USA) for 24 h prior to staining, lysing, and quantifying cell migration according to kit instructions. Within the Cell Migration Assay, cells have to migrate through an 8 mm pore membrane to a bottom chamber. Invaded cells on the bottom of the insert membrane are dissociated from the membrane when incubated with Cell Detachment Buffer. These cells are subsequently lysed and detected by the patented CyQUANT GR dye (Molecular Probes, Invitrogen, Carlsbad, CA, USA). This green-fluorescent dye exhibits strong fluorescence enhancement when bound to cellular nucleic acids Quantification (i.e., cell migration) was assessed by fluorescence readings using a Tecan Infinite M200 Pro (Tecan Group, Männedorf, Switzerland) plate reader. Samples were run in biological triplicate with multiple reads per well.

### 2.8. Statistical Analysis

A one-way ANOVA test with Bonferroni’s multiple comparison analysis was used to compare the mock and treatment groups for the viral titers, cytokines and cell migration. All tests were conducted in Prism (GraphPad, La Jolla, CA, USA) v5. Significance of gene expression from the RT-qPCR arrays was determined by paired *t*-test performed in Microsoft Excel 2013 (Microsoft, Redmond, WA, USA). Hierarchical clustering and heatmap generation was performed using Mathematica v11 (Wolfram, Champaign, IL, USA).

## 3. Results

The goal of this study was to determine if human dermal APCs are susceptible to infection by CCHFV and to determine the impact of tick saliva on infected APCs. We were able to generate the human dermal APCs, dermal dendritic cells (dDC) and Langerhans cells (LC), from umbilical cord blood donors in vitro with high purity (>90%) as previously described [25]. In order to evaluate the influence of tick saliva on the susceptibility and immune response of the APCs, salivary gland extract (SGE) was generated from uninfected *Hyalomma marginatum* ticks on the second days after feeding commenced. Size and concentration of protein fractions in SGE can be found in Supplementary Figure S1.

Both LCs and dDCs were susceptible and permissive (~3% and ~25%, respectively) to infection with CCHFV prototype strain IbAr102000 (Figure 1A). The viral output ranged between 31 and 80,000 PFU/mL 48 h post infection. Although there was noteworthy donor to donor variation in viral output, there was a significant difference in viral titers in supernatant between dDCs and LCs. This is especially interesting since the dDCs and LCs are derived from the same donor. Surprisingly, SGE did not significantly influence the viral output in either of the two cell types. The same findings were found with a different CCHFV strain AP92/P7 (Figure 1B). This strain was isolated from a region with observed human seroprevalence but no recorded disease. This has led to speculation that AP92/P7 might be a low-virulence strain of CCHFV and therefore might display differences between the prototype strain (IbAr10200). However, the AP92/P7 strain grew to similar titers, ranging from 15 to 30,000 PFU/mL^−1^ 48 h post infection.

**Figure 2:**
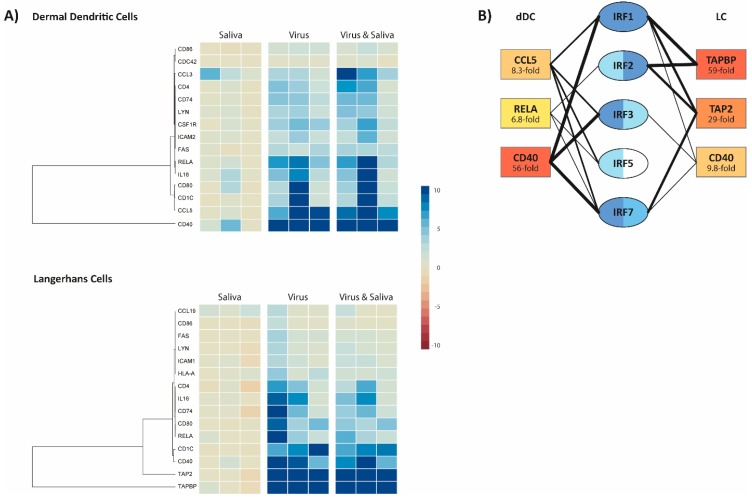
Expression of antigen-presentation genes post-CCHFV infection and/or SGE stimulation. Human dDCs and LCs were stimulated with SGE and/or infected with CCHFV (strain IbAr 10200). After 12 h, whole RNA was prepared from the cells for RT-qPCR analysis. Significantly altered genes were determined for each cell type, with fold-changes clustered using hierarchical clustering (**A**). The three most-altered genes for each cell type were used to predict upstream transcription factor activation using Ingenuity Pathway Analysis (**B**). Potential transcription factor involvement (blue highlighting) was predicted based upon significance of interaction between the factor and the highly-upregulated gene, and the strength of gene upregulation, with darker blue highlighting indicating a greater likelihood of involvement. Intensity of connecting lines are qualitative based upon the fold changes of the associated genes.

Differentiated dDCs and LCs from three donors were stimulated with tick saliva and/or infected with CCHFV IbAr 10200 at a MOI of 0.1. After 12 h, total RNA was harvested for RT-qPCR array analysis. Significantly-altered genes were determined for each cell type, and expression profiles were grouped by hierarchical clustering (Figure 2A). Substantial variability in gene expression was observed between donors, particularly in the dDCs. In general, infection with CCHFV led to the increased expression of most of the significantly-altered genes. Moreover, the fold-changes were often greater in the LCs than in the dDCs.

In order to determine the potential upstream transcription factors responsible for the observed differences in gene expression between dDCs and LCs, the three most substantially altered genes for each cell type were analyzed using Ingenuity Pathway Analysis. This revealed that expression of all these factors involves the activation of Interferon Regulatory Factors (IRFs; Figure 2B). The high level of expression of Tapasin Binding Protein (TAPBP) and Antigenic Peptide Transporter 2 (TAP2) (59-fold and 29-fold average expression, respectively) in virus-infected and virus-infected, saliva-stimulated LCs (mean fold changes for both conditions), with lower expression of other factors (<10-fold increase), suggests strong interferon regulatory factor (IRF)-1 activation. By contrast, the similarly high level of expression of CD40 (56-fold) in virus-infected and virus-infected, saliva-stimulated dDCs (mean fold changes for both conditions), with lower expression of other factors (<10-fold increase), suggests strong IFR-7 activation.

Interestingly, stimulation of the cells with tick saliva had minimal effect on the transcription profiles of the cells. In general, addition of saliva alone led to minor, general downregulation (<2-fold), although in dDCs the addition of saliva led to a slight increase in CCL3 expression. Moreover, gene expression profiles in dDCs and LCs following virus infection and virus infection with addition of tick saliva were very similar. The exceptions to this are CCL3 and CD4 expression in dDCs, where the addition of tick saliva enhanced virus-associated gene expression.

**Figure 3:**
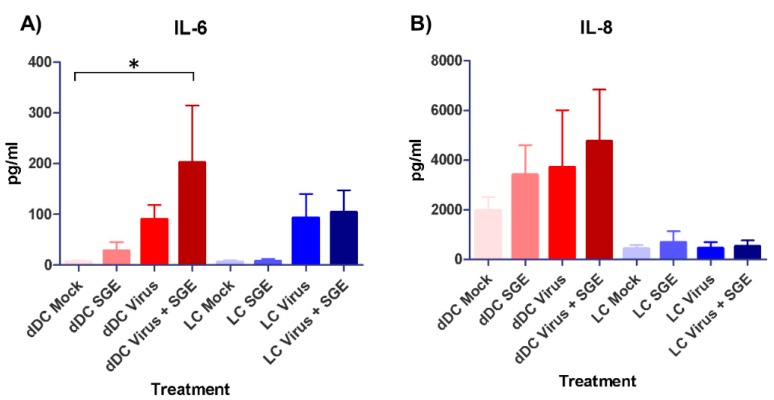

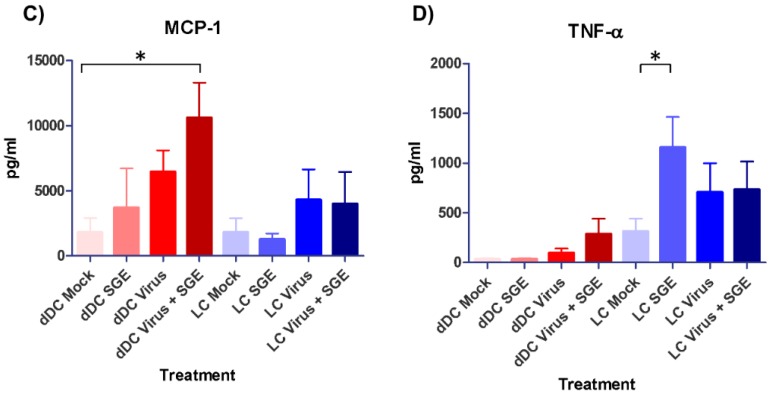
Cytokine and chemokine responses of mock and treated skin APCs. Human dDC and LC were studied using a multiplexed antibody/analyte detection assay. Cytokines (**A**,**D**) and chemokines (**B**,**C**) were detected by harvesting mock and treatment cell (*n* = 5) clarified supernatants at 24 hpi after infection with CCHFV (strain IbAr 10200) and treatment with or without SGE; and run in technical duplicate on a Luminex bead-based multiplex system with kit provided control standards for pg/mL determinations. Significance between mock and indicated treatment groups at *p* < 0.05 is designated with an asterisk (*) symbol.

Cytokine and chemokine responses of mock and treated human dDC and LC were studied using a multiplexed detection assay on clarified cell supernatants at 24 hpi. Of the 13 tested cytokines and chemokines, only four showed substantial changes when compared to the mock and/or between the cell types. As previously described, the cytokine and chemokine secretion profile of the two cell types was different [29]. Statistically significant increases in the secretions of cytokine IL-6 and chemokine MCP-1 were observed in dDC supernatants that were both infected with CCHFV and treated with SGE in terms of mock comparisons (Figure 3A,C). LC supernatants demonstrated significant increases in the secretion of cytokine TNF-α only in SGE treated cells compared to mock (Figure 3D). Higher levels of chemokine secretions (IL-8 and MCP-1) were detected among all treatment groups in dDCs compared to LC treatment groups (Figure 3B,C).

**Figure 4:**
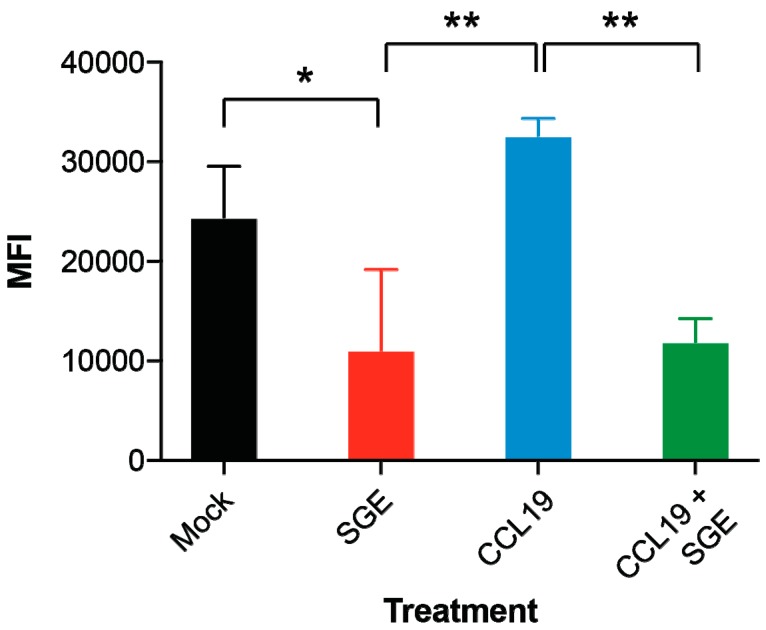
Salivary gland extract from *Hyalomma* ticks inhibits migration of human monocyte-derived dendritic cells (moDC). moDC migration was studied in a trans-well migration assay. When moDC where exposed to salivary gland extract (SGE) fewer cells migrated to the other side of the trans-well (mock = RPMI only) or with chemoattractant (CCL19). Number of migrated cells was assessed by lysing cells and analyzing DNA content by intercalating dye. Fluorescent was measured as mean fluorescent intensity (MFI). Significance between mock and indicated treatment groups at *p* < 0.05 and *p* < 0.01 is designated with one or two asterisk (*) symbols, respectively.

We noticed that dDCs and LCs exposed to *Hyalomma* tick SGE with or without virus started to aggregate in clusters raising the question of whether the cells lose their adhesiveness necessary to leave the tissues. Other studies have shown that tick saliva can inhibit the migration of dendritic cells [28]. Cell migration is a fundamental function of APCs’ immune response and in triggering of inflammation cascades. An analysis of the expression of the migration marker CD197 indicated that marker is expressed when APCs are infected with CCHFV, however, expression levels remain similar to mock when SGE is added. To study the effects of *Hyalomma* tick SGE on APCs migration, we employed a system for quantitative determination of numerous factors on cell migration using pharmacological agents, similar to Boyden chamber migration assays. Using serum starved conditions, we exposed monocyte-derived dendritic cells (moDC) to SGE, CCL19 (chemoattractant for APCs), and both SGE and CCL19. SGE treated cells alone inhibited migration across chambers with a statistically significant decrease compared to mock (Figure 4). Chambers containing CCL19, demonstrated increase in cell amounts (MFI) of moDC into chambers, indicating enhanced/increased migration due to chemotaxis (Figure 4). However, SGE treated moDC when exposed to chambers containing CCL19 had a statistically significant decrease in the number of observed cells migrated into the CCL19 chamber, indicating SGE inhibited chemotactic migration of these moDC (Figure 4).

## 4. Discussion

Previous studies have shown that DCs and macrophages are the most likely target cells for CCHFV upon subcutaneous introduction by tick bite [7,8]. Here, we expand this to dermal APCs, and demonstrate that LCs and dDCs are susceptible and permissive to CCHFV infection and replication. There was noteworthy donor to donor variation, suggesting a potential genetic influence on the infectivity, however, mean values of titers of the different groups stayed within the same range. Interestingly, there was a significant difference in terms of post-infection CCHFV titers between dDCs and LCs. The fact that the two cell types were derived from the same donor suggested that the differentiation state of the cell type might have an influence on the permissivity. A recent study looking at Ebolavirus sets a comparable precedent where monocytes and macrophages have different permissivities [10]. Interestingly, *H. marginatum* SGE did not enhance the viral output in either of the cell types. In this study we only evaluated SGE from semi-engorged *Hyalomma marginatum* ticks at a concentration of 10 μg. Furthermore, SGE was mixed with the virus and cells were not pretreated. It is conceivable that modifying the parameters of the experimental setup by harvesting SGE at a different time point, pretreatment, or using a different concentration could have different effects on the viral output. Nevertheless, we tried to mimic the natural conditions as best as possible in our experimental design. The findings mentioned above also held true when a different CCHFV strain, AP92/P7, was used. AP92/P7 is assumed by some to have low virulence based on indirect epidemiological evidence [30,31]. Although CCHFV AP92/P7 titers in dDC and LC were not statistically different than CCHFV IbAr10200 titers, additional studies are needed to identify the virulence level of the strains.

Our gene array results indicate that the response to CCHFV in the two cell types is potentially mediated through different Interferon Regulatory Factors (IRF). IRFs are key transcription factors in the cellular response to viral infections. Previous studies have demonstrated that they play significant roles in CCHFV infection, with viral inhibition of IRF3 through the ovarian tumor (OTU) domain of the viral polymerase [32]. Our studies suggest that in dDCs, IRF7 is primarily activated following infection with CCHFV, whereas in LCs, IRF1 activation is primarily increased. Recent studies by Feng et al. (2018), have demonstrated that while both IRF1 and IRF7 decrease CCHFV production when overexpressed in Vero cells, the inhibitory effect of IRF1 is greater [33]. This fits with our data, as the LCs appear to have a stronger IRF1 activation, and lower viral titers, whereas the dDCs appear to have a stronger IRF7 activation, and higher titers. Owens et al. has shown that a IRF7 knock-out result in DC hyper-activation [34], suggesting that IRF7 may have an additional role in moderating DC activation and antiviral responses. Unfortunately, we did not include a group of cells treated with just the inactivated virus, which could have given an assessment if the stimulation of dDCs and LCs depends on viral replication or is just caused by viral binding and update. Moreover, the sample volumes were too small to be able to directly determine the activation states of the IRFs.

Cytokines and chemokines are crucial signaling proteins in the pathogenesis of viral diseases with functions such as immunomodulation and attracting cells to sites of infection. In this study, we observed that both dDCs and LCs secrete high levels of IL-6 and MCP-1 when infected with CCHFV with or without SGE. Nevertheless, the cytokine/chemokine profile was not always the same for dDC and LC, with IL-8 secreted to higher levels in dDC, and TNF-α higher in LCs, most likely reflecting the different functions of the cells. Our findings for cytokine/chemokine secretion of dDCs falls between the findings of Connolly-Andersen et al. [8], showing that DCs produce high amounts of IL-6 and TNF-α but not IL-8, and Peyrefitte et al. [7], detecting high amounts of IL-6 and IL-8 but not TNF-α. Although the MOI was different between the studies, it is conceivable that the differentiation state of dDCs leads to secretion of a different cytokines/chemokine profile compared to the monocyte-derived DC used by Connolly-Andersen et al. and Peyrefitte et al. SGE alone induced high levels of TNF-α secretion in LCs, however, overall lead to only minor changes in cytokine/chemokine secretion. Interestingly, SGE and CCHFV had synergistic effects on IL-6, MCP-1 and TNF-α secretion. High levels of chemokines and pro-inflammatory cytokines will attract mononuclear host cells to the tick feeding site. Some of these cells, such as macrophages and DCs, are target cells for CCHFV and therefore serve the virus as an additional replication site. An aberrant cytokine secretion induced by CCHFV and SGE might also lead to an inadequate stimulation of the adaptive immune response [35].

Tick saliva in general poses a wide range of immunomodulatory functions [18,36,37], and on top of cytokine/chemokine perturbations, tick saliva can also impact antigen presenting cell maturation factors [37]. It is still unclear what specific factors impact maturation, however, in a maturation impeded state, APCs may fail to upregulate cell-surface receptors involved in chemotaxis/ligand binding to inducing homing to draining lymph nodes. To study if *Hyalomma* tick saliva could also impact this type of homing, we used the human chemokine CCL19 to induce migration in activated APCs that were either treated or untreated with *Hyalomma* SGE. Our results reinforce what others have shown for other ixodid tick salivas [28,37], which is that *Hyalomma* can also impede APCs migration and chemotaxis through salivary compounds. It would be fascinating to develop and test a luciferase-labeled CCHFV for in vivo imaging, of an infected tick bite onto an animal. This would be useful to determine if our data regarding migration, could be proven in situ of the tick bite/animal interface.

## 5. Conclusions

Our findings indicate that human dDCs and LCs are susceptible and permissive to CCHFV infection, however, to different degrees. Infection leads to cell activation and cytokine/chemokine secretion. *Hyalomma marginatum* SGE only had little effect on the cells, with some synergy of viral infection when it comes to cytokine secretion. Based on the findings here, we assume that tick saliva does not necessarily have an influence on CCHFV replication, but rather potentially inhibits migration of APCs and immunomodulates the feeding environment.

## Supplementary Materials

Supplementary materials can be found at http://www.mdpi.com/1999-4915/10/7/381/s1.
