# Supplementary material for: Interactions of Human Dermal Dendritic Cells and Langerhans Cells Treated with Hyalomma Tick Saliva with Crimean-Congo Hemorrhagic Fever Virus
Source: Viruses. 2018 Jul 20;10(7):381. doi: 10.3390/v10070381 (PMC6070959; doi:10.3390/v10070381)
Supplement: Supplementary file 1 [file viruses-10-00381-s001.pdf]

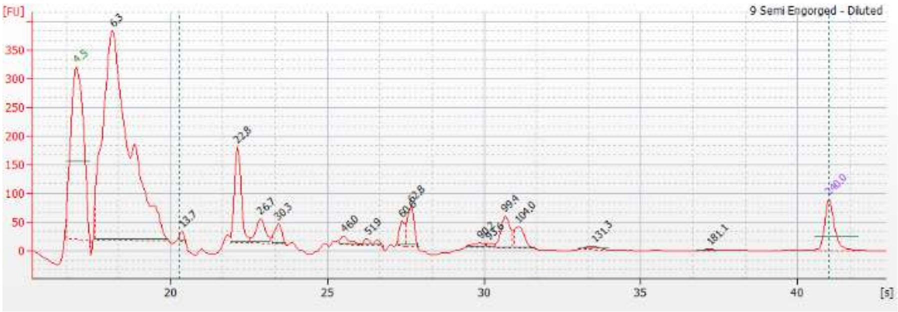

Supplementary figure 1. Size and concentrations of protein fractions in *Hyalomma marginatum* salivary extract. SGE was generated from ticks on the second day post attachment. SGE was run on BioAnalyzer using a Agilent Protein 80 Kit.
